# Analysis of gut microbiota-derived metabolites regulating pituitary neuroendocrine tumors through network pharmacology

**DOI:** 10.3389/fphar.2024.1403864

**Published:** 2024-09-04

**Authors:** Min Cao, Ping Huang, Lun-shan Xu, Yi-hua Zhang

**Affiliations:** Department of Neurosurgery, Daping Hospital, Army Medical University, Chongqing, China

**Keywords:** pituitary neuroendocrine tumors, gut microbiota, metabolites, tryptophan metabolism, CXCL8

## Abstract

Pituitary neuroendocrine tumors (PitNETs) are a special class of tumors of the central nervous system that are closely related to metabolism, endocrine functions, and immunity. In this study, network pharmacology was used to explore the metabolites and pharmacological mechanisms of PitNET regulation by gut microbiota. The metabolites of the gut microbiota were obtained from the gutMGene database, and the targets related to the metabolites and PitNETs were determined using public databases. A total of 208 metabolites were mined from the gutMGene database; 1,192 metabolite targets were screened from the similarity ensemble approach database; and 2,303 PitNET-related targets were screened from the GeneCards database. From these, 392 overlapping targets were screened between the metabolite and PitNET-related targets, and the intersection between these overlapping and gutMGene database targets (223 targets) were obtained as the core targets (43 targets). Using the protein–protein interaction (PPI) network analysis, Kyoto encyclopedia of genes and genomes (KEGG) signaling pathway and metabolic pathway analysis, CXCL8 was obtained as a hub target, tryptophan metabolism was found to be a key metabolic pathway, and IL-17 signaling was screened as the key KEGG signaling pathway. In addition, molecular docking analysis of the active metabolites and target were performed, and the results showed that baicalin, baicalein, and compound K had good binding activities with CXCL8. We also describe the potential mechanisms for treating PitNETs using the information on the microbiota (*Bifidobacterium adolescentis*), signaling pathway (IL-17), target (CXCL8), and metabolites (baicalin, baicalein, and compound K); we expect that these will provide a scientific basis for further study.

## 1 Introduction

Pituitary neuroendocrine tumors (PitNETs) constitute a special class of tumors of the central nervous system that are closely related to metabolism, endocrine functions, and immunity (Nie et al., 2023). A recent epidemiological study showed that PitNETs affect more than 5% of the global population (Wang et al., 2023a). Intestinal flora interact with the central nervous system via the gut–brain axis, which includes the vagus nerve, enteric nervous system, immune system, and microbial metabolites. Gut microbiota are closely related to the functions of the central nervous system as well as the endocrine and neuroendocrine systems (Leistner and Menke, 2020; Tafet and Nemeroff, 2020; Wang et al., 2023b; Berding et al., 2021; Longo et al., 2023), but their relationships with PitNETs are not completely clear.

Research has shown that the gut microbiomes of patients with PitNETs are significantly different from those of healthy subjects. Compared with the control group, *Clostridium innocuum* was enriched while *Oscillibacter* sp.57_20 and *Fusobacterium mortiferum* populations were poorer in patients with invasive and non-invasive PitNETs (Hu et al., 2022). Another study showed that there were differences in the structures and quantities of intestinal flora among growth-hormone-secreting PitNET patients, non-functional PitNET patients, and healthy controls. In the mouse model, following transplantation of fecal microflora, the intestinal flora of growth-hormone-secreting PitNETs patients promoted the growth of tumor (Nie et al., 2022). Another study showed that compared with healthy controls, growth-hormone-secreting PitNETs not only reduce the alpha diversity of the intestinal flora but also change the beta diversity significantly (Lin et al., 2022). Among the metabolites, the short-chain fatty acid butyrate was shown to enhance growth hormone secretion in the rat anterior pituitary cells through activation of the G-protein-coupled receptors GPR41 and GPR43 (Miletta et al., 2014). These studies indicate that there are potential relationships among PitNETs, intestinal flora, and metabolites. Although some of these relationships have been reported in literature, their specific mechanisms and action pathways need to be clarified through further research. Network pharmacology can comprehensively consider the interactions between metabolites and multiple targets, thereby offering more insights into the comprehensive effect mechanisms (Oh et al., 2023).

**FIGURE 1:**
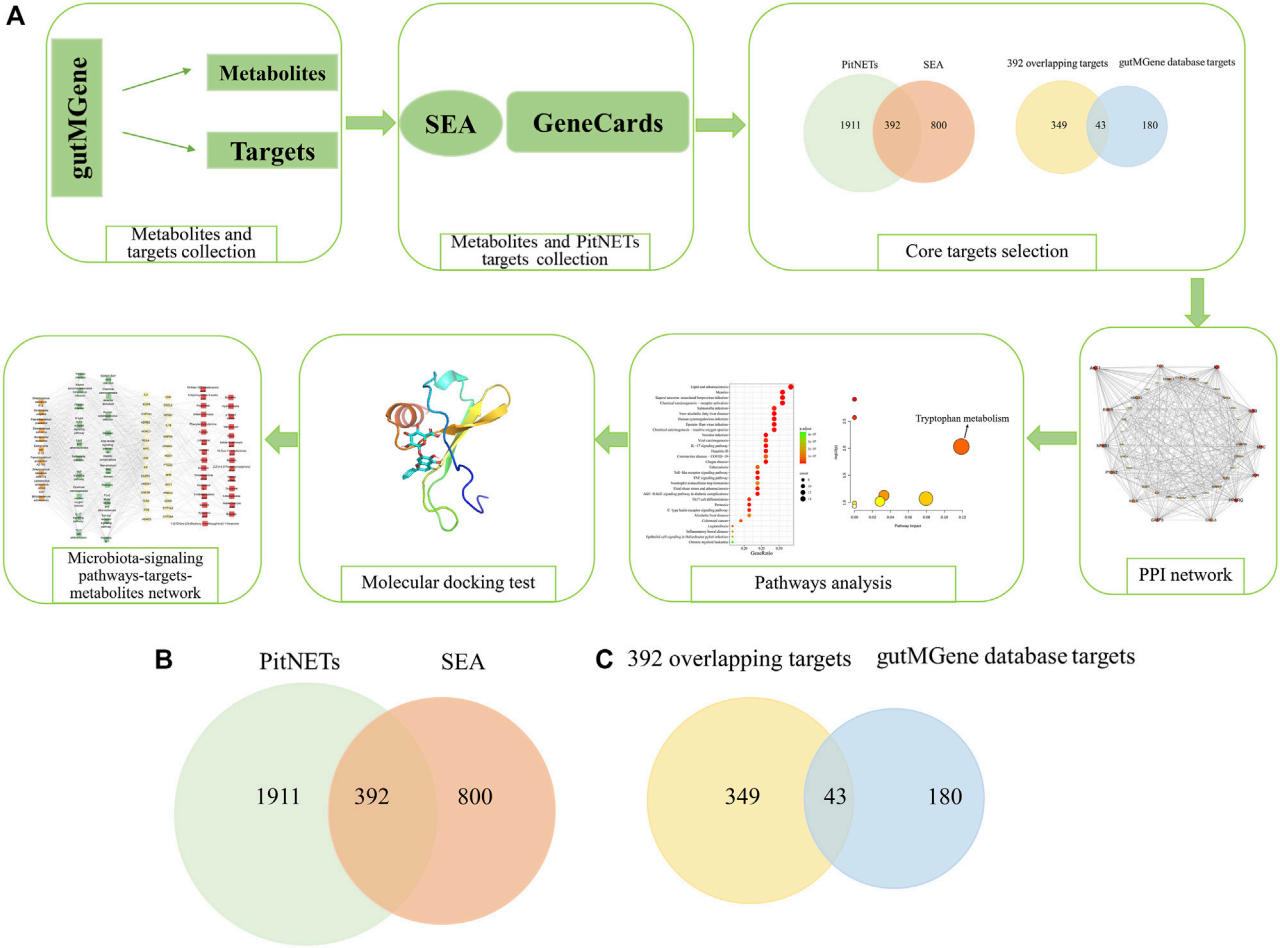
**(A)** Workflow of the steps in this study. **(B)** Overlapping targets (392) between metabolite targets predicted by the similarity ensemble approach (SEA) database and PitNET-related targets. **(C)** Identification of 43 core targets between the 392 overlapping targets and gutMGene database (223 targets).

In the present study, the key targets, signaling pathways, metabolites, and microbiota that regulate PitNETs were identified via network pharmacology, and their relationships were revealed. First, the gutMGene v1.0 database (http://bio-annotation.cn/gutmgene/) (Cheng et al., 2022) was used to retrieve the metabolites and targets related to the gut microbiota; then, the metabolite-related and PitNET-related targets were determined through public databases. Second, the overlapping targets between the metabolite-related and PitNET-related targets were obtained via Venn diagram; these overlapping targets were further intersected with the targets obtained from the gutMGene database via a Venn diagram to determine the final common targets. These final overlapping targets were considered as the core targets. Third, the protein–protein interaction (PPI) network and Kyoto encyclopedia of genes and genomes (KEGG) pathway enrichment diagram were constructed using the core targets; the metabolic pathways were then analyzed using the corresponding metabolites of the core targets to identify the key signaling pathways and targets in PitNETs. Once the key target was identified, molecular docking analysis was used to predict the potential metabolites that acted on the key target. Finally, the relationships among the identified microbiota, signaling pathways, targets, and metabolites were examined to reveal their roles in PitNETs. Therefore, this study is expected to be an important reference for further experimental research and regulation of metabolites with regard to PitNETs. The steps in the workflow of this study are shown in Figure 1A.

## 2 Methods

### 2.1 Data collection and target acquisition

The metabolites and targets used in the analyses were retrieved from the gutMGene v1.0 database. The related targets of the metabolites were predicted using the similarity ensemble approach (SEA) (https://sea.bkslab.org/) (Keiser et al., 2007) database. The disease targets were obtained from the GeneCards database (https://www.genecards.org/) (Safran et al., 2010), and targets that were larger than the median were selected for subsequent analyses. The intersection of metabolite targets and disease targets were obtained by Venn analysis. Finally, the core targets were obtained from the intersection of all targets from the three databases; we believe that these core targets are important factors in PitNETs.

### 2.2 Protein–protein interaction network

The core targets were mapped to the PPI network to identify their interactions. The STRING database (https://string-db.org/) was used to perform the PPI analysis.

### 2.3 KEGG pathway and gene ontology (GO) analyses

R package (Yu et al., 2012) was used to perform KEGG pathway enrichment (Draghici et al., 2007) and GO analysis to analyze targets functions (Luo et al., 2017). Furthermore, the interactions between the key regulatory pathways were analyzed using R software.

### 2.4 Metabolic pathway analysis

MetaboAnalyst 6.0 (https://www.metaboanalyst.ca/) was used for the metabolic pathway analysis. Here, 31 metabolites corresponding to the 43 core targets were input into the online website for the pathway analysis.

### 2.5 Molecular docking test

The structured data files of the metabolites were acquired from the PubChem database (https://pubchem.ncbi.nlm.nih.gov/), and the target protein structure was downloaded from the PDB database (https://www1.rcsb.org/). The ligands and protein needed for molecular docking analysis were prepared using AutoDock, and the molecular docking was carried out using Vina in PyRx. The docking results were finally visualized using PyMOL software.

### 2.6 Construction of the microbiota-signaling pathways-targets-metabolites network

Based on the KEGG enrichment results of the 43 core targets, the top-20 significant signaling pathways were determined as the main signaling pathways. Then, the microbiota and metabolites that were directly related to these top-20 pathways’ targets were selected using the gutMGene database. We chose the top-10 results based on the degree of microbiota to construct the microbiota–signaling pathways–targets–metabolites network, which was then visualized using Cytoscape 3.10.1 (Shannon et al., 2003).

## 3 Results

### 3.1 Core targets of the gut microbial metabolites

A total of 208 metabolites were obtained from the gutMGene database. The targets related to these metabolites were identified using the SEA database, through which 1,192 metabolite targets were found. A total of 392 overlapping targets were then identified between the 1,192 metabolite targets and 2,303 PitNET-related targets, as shown in Figure 1B. Finally, the intersection between these 392 overlapping targets and 223 targets from the gutMGene database yielded 43 core targets that were used in the follow-up analyses (Figure 1C) (Supplementary Table S1).

### 3.2 Protein–protein interaction network analysis

**FIGURE 2:**
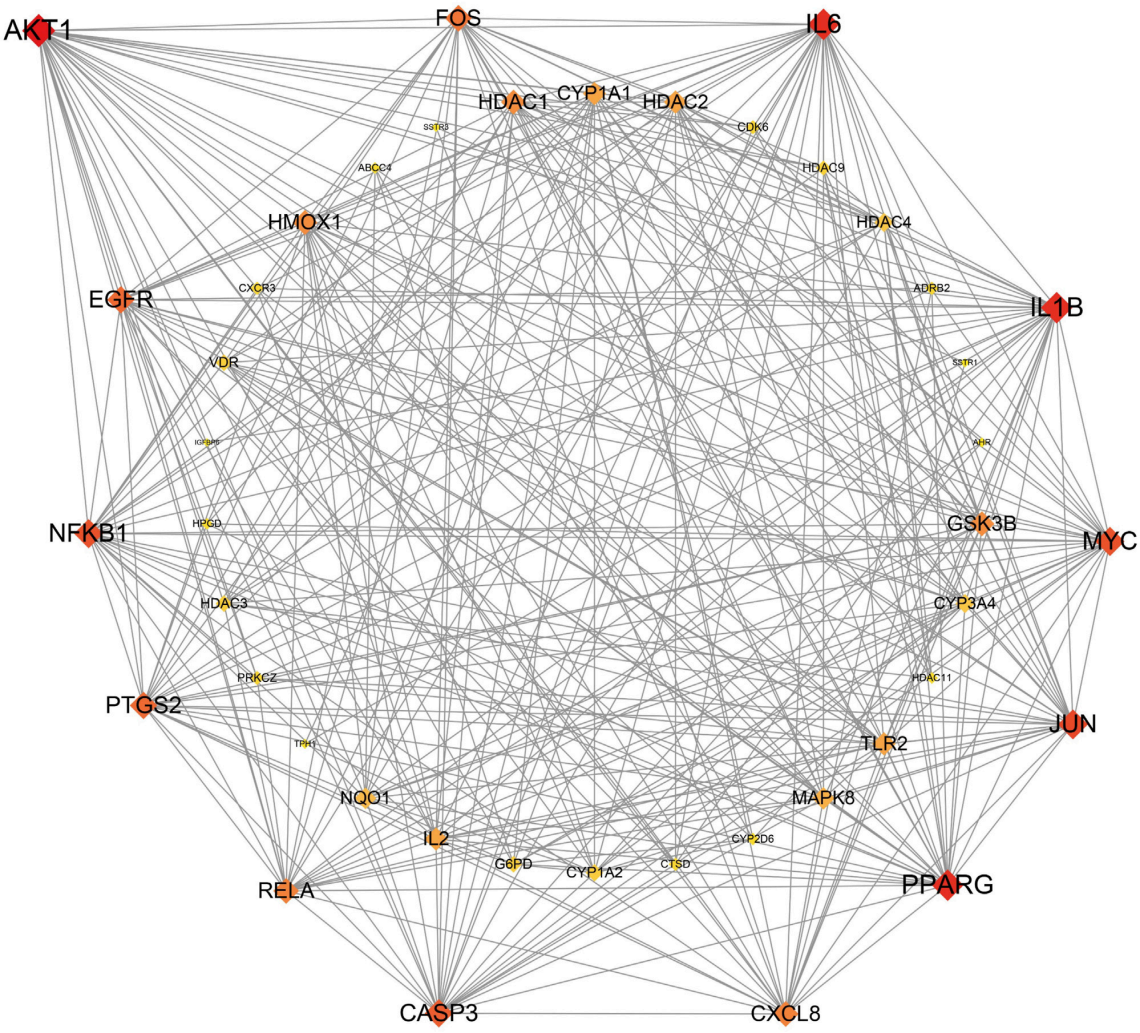
Protein–protein interaction network in this study.

The 43 core targets were used in the PPI network analysis to highlight the important proteins. The protein AKT1 has the highest degree of 31, indicating that it may be an important target related to PitNETs. To date, many studies have shown that AKT1 is closely related to PitNETs (Huang et al., 2020; Dworakowska et al., 2009). Therefore, our findings are consistent with those reported in previous studies. In the present study, the top-13 proteins based on degree are AKT1 (31), IL1B (29), IL6 (29), PPARG (29), JUN (27), MYC (26), NFKB1 (26), CASP3 (25), EGFR (24), PTGS2 (24), FOS (23), CXCL8 (22), and RELA (22) (Figure 2), indicating that these proteins may be related to PitNETs.

### 3.3 Signaling pathway and GO enrichment analyses

**FIGURE 3:**
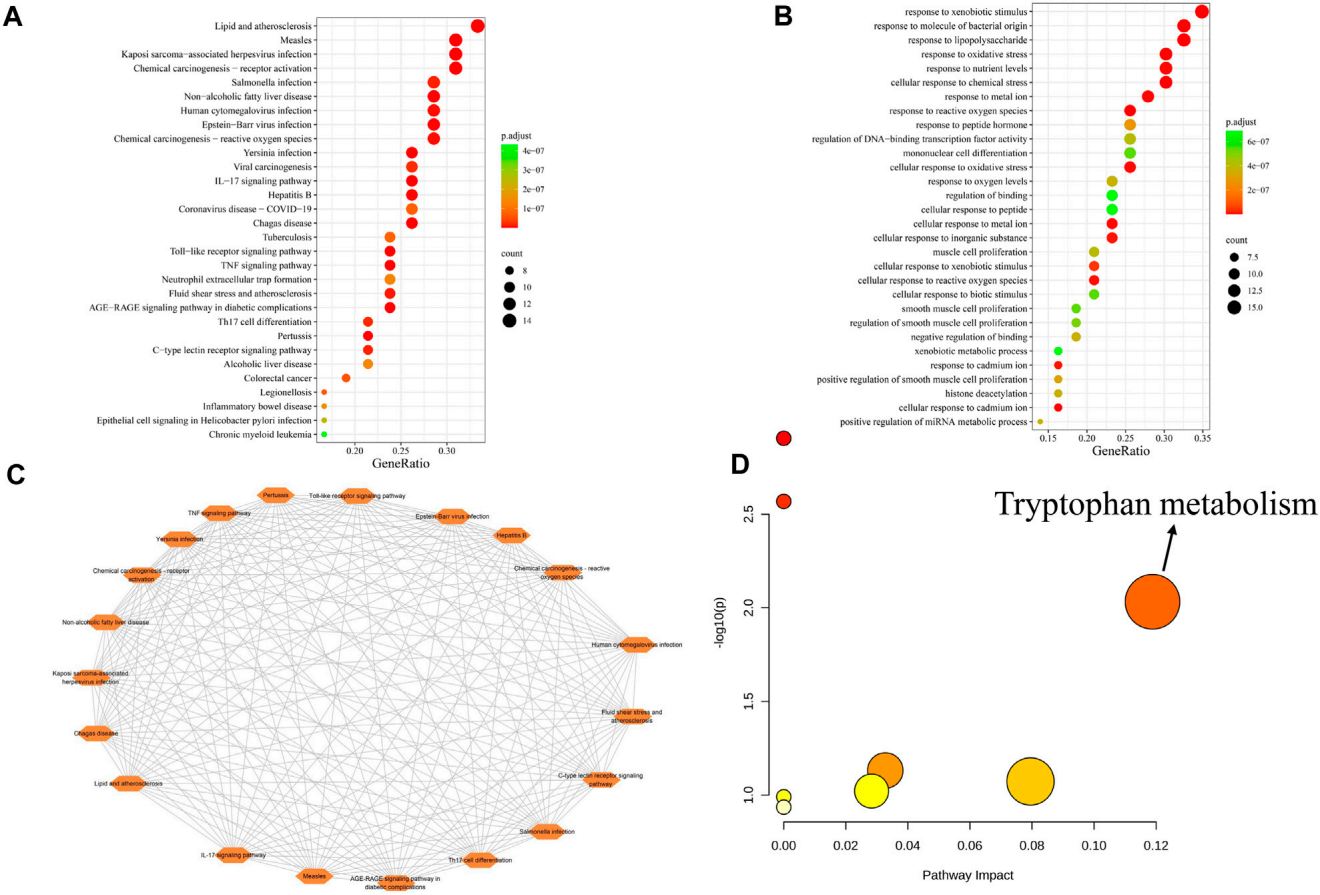
**(A)** KEGG enrichment analysis of the 43 core targets. **(B)** GOBP enrichment analysis of the 43 core targets. **(C)** Pathway interaction diagram. **(D)** Metabolic pathway analysis of the 31 metabolites identified from the 43 core targets. The colors of the dots represent their *p*-values from the pathway enrichment analyses; color variation from yellow to red indicates that the *p*-value is decreasing. The size of the dot represents the pathway impact value from the pathway topology analysis, where the larger the dot, the greater the pathway impact.

The 43 core targets were used in the KEGG (Figure 3A) and GO (Figure 3B) enrichment analyses using R programming language. The KEGG results showed that these pathways related to these targets mainly involved inflammation, immune responses, infection, and cancer. The IL-17 signaling pathway is related to inflammation (Amatya et al., 2017), and inflammation has been observed to be closely related to the development of PitNETs (Wang et al., 2021; Chen et al., 2015); and the IL-17 signaling pathway (has04657) has a significant enrichment effect. Studies have shown that IL-17 is a proinflammatory cytokine that promotes the growth and progression of cancer by activating various signaling pathways (Inthanon et al., 2023). In addition, the serum IL-17 levels in patients with invasive PitNETs are significantly higher than those in patients with non-invasive PitNETs (Qiu et al., 2011); the median serum IL-17A level in patients with PitNETs is higher than that in the control group (Glebauskiene et al., 2018). This suggests that the IL-17 signaling pathway may play an important role in the development and invasive behaviors of PitNETs. The GO enrichment analysis results showed that biological processes were related to immune and inflammatory responses, oxidative stress, antioxidant responses, and cell proliferation and differentiation. We further analyzed the interactions between the top-20 significant regulatory pathways (Figure 3C), whose interaction degrees were all equal to 19. Furthermore, 31 metabolites corresponding to the 43 core targets were input into MetaboAnalyst 6.0 for the metabolic pathway analysis (Figure 3D), and pathway impacts exceeding 0.1 were selected as the potential metabolic pathways. In this study, only one potential metabolic pathway of tryptophan metabolism was observed to be enriched. A recent study shown that tryptophan metabolism was closely related to tumor growth (Liu et al., 2023). Based on the above results and published literature, it was found that tryptophan metabolism was related to the protein CXCL8. Tryptophan metabolizing enzymes can accelerate tumor progression by upregulating CXCL8 (Zhao et al., 2021), which is also a chemokine in the IL-17 signaling pathway, and CXCL8 has a high degree value in PPI network; hence, we speculate that CXCL8 could be a potential target in the treatment of PitNETs.

### 3.4 Molecular docking analysis

**FIGURE 4:**
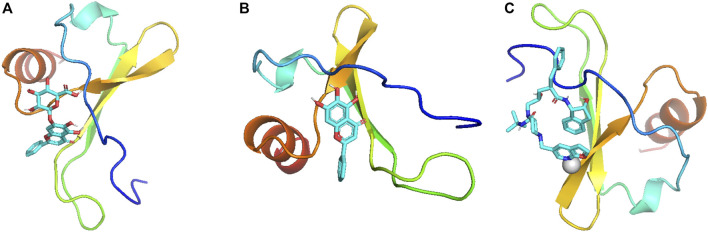
Molecular docking visualization of **(A)** baicalin–CXCL8, **(B)** baicalein–CXCL8, and **(C)** compound-K–CXCL8.

Molecular docking was used to evaluate the binding energies of CXCL8 (PDB ID: 6WZM) with six potentially related metabolites from the gutMGene database. These metabolites included succinate; acetate; 1-piperazinepentanamide, N-((1S,2R)-2,3-dihydro-2-hydroxy-1H-inden-1-yl)-2-(((1,1-dimethylethyl)amino)carbonyl)-4-(furo(2,3-b)pyridin-5-ylmethyl)-gamma-hydroxy-alpha-(phenylmethyl)-, (alphaR, gammaS,2S)- (also known as compound K); butyrate; baicalein; and baicalin. When the binding energy for docking is less than −5 kcal/mol, it indicates that there is good binding activity between the protein and metabolite. The results showed that the binding energy of CXCL8 with baicalin was −7.0 kcal/mol (Figure 4A), that with baicalein was −6.3 kcal/mol (Figure 4B), and that with compound K was −5.8 kcal/mol (Figure 4C) (Supplementary Table S2). The binding energies of CXCL8 with the other three metabolites exceeded −5 kcal/mol. These results show that baicalin, baicalein, and compound K may be potential metabolites affecting PitNETs.

### 3.5 Analysis of the microbiota-signaling pathways-targets-metabolites network

**FIGURE 5:**
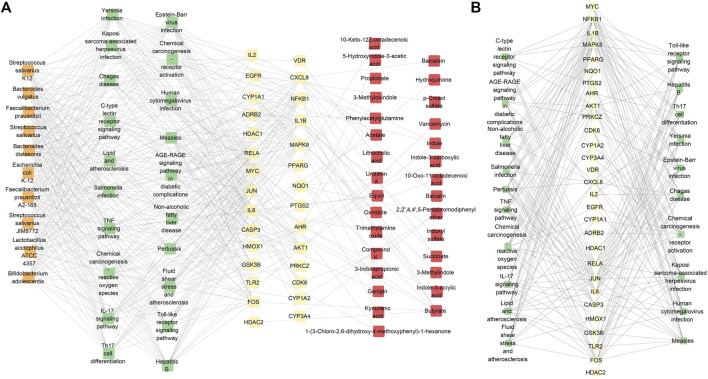
**(A)** Proposed microbiota-signaling pathways-targets-metabolites network. To show the relationships between the microbiota and pathways more clearly, only the top 10 microbiota are shown based on their interaction degrees. **(B)** Network showing the signaling pathways and targets.

The microbiota and metabolites directly related to the top-20 significant signaling pathway targets were selected from the gutMGene database. In this study, we only selected the top-10 results based on the degree of microbiota for subsequent analyses. The microbiota–signaling pathways–targets–metabolites network consisted of 89 nodes (10 microbiota, 20 signaling pathways, 29 targets, and 30 metabolites) and 479 edges (Figure 5A). To show the relationships between the signaling pathways and targets more clearly, we indicate the relationships between the signaling pathways and targets in the network separately (Figure 5B). Here, the orange boxes represent the gut microbiota, green boxes represent the signaling pathways, yellow boxes represent the targets, and red boxes represent the metabolites. We found that these top-10 microbiota had the same interaction degrees: *Bifidobacterium adolescentis*, *Escherichia coli* K-12, *Streptococcus salivarius* K12, *Streptococcus salivarius* JIM8772, *Lactobacillus acidophilus* ATCC 4357, *Faecalibacterium prausnitzii*, *Streptococcus salivarius*, *Bacteroides distasonis*, *Bacteroides vulgatus*, and *Faecalibacterium prausnitzii* A2-165. Studies have found that *B. adolescentis* displays distinct anti-inflammatory effects (Guo et al., 2019) and can suppress tumorigenesis (Lin et al., 2023; Chen et al., 2023). Previous studies have also shown that inflammation is closely related to the development of PitNETs (Wang et al., 2021; Chen et al., 2015). Therefore, we speculate that *B. adolescentis* may be an important microbe related to PitNETs. These findings, along with the results of our previous research, show that tryptophan metabolism could be a potential metabolic pathway and could be related to CXCL8. The molecular docking analysis results show that baicalin, baicalein, and compound K have good binding activities with CXCL8 that participates in the IL-17 signaling pathway; furthermore, the IL-17 signaling pathway is related to inflammation, and *B. adolescentis* plays an important role in inflammatory response. Thus, in the microbiota–signaling pathways–targets–metabolites network, we found that baicalin, baicalein, and compound K were potentially related to CXCL8, the IL-17 signaling pathway, and *B. adolescentis*. These results show that these six elements could be used as potential markers in the treatment of PitNETs.

## 4 Discussion

PitNETs are commonly divided into functional and non-functional PitNETs. Functional PitNETs can be further divided into various types based on the secretion of hormones, among which prolactinoma is the most common, followed by growth hormone and adrenocorticotropic hormone. We screened the 43 core targets related to PitNETs from the gutMGene v1.0 database and noted that 31 key metabolites corresponding to the 43 core targets were enriched in the tryptophan metabolism pathway. A prior study reported that prolactinoma was related to tryptophan metabolism (Zhou et al., 2015). The change in amino acid metabolism has been considered as a characteristic of tumor cells, which significantly impact the tumor cells and the immune regulation mechanisms in the tumor microenvironment (Xu et al., 2021). Tryptophan is an essential amino acid and a precursor to serotonin; serotonin is a key neurotransmitter in the enteric system and central nervous system. Gut microbiota can potentially affect the brain and behaviors by controlling the availability of the serotonin precursor tryptophan. Gut microbiota also indirectly controlled the hypothalamic–pituitary–adrenal axis (Clarke et al., 2014). This suggests that tryptophan metabolism may play a key role in the development of PitNETs and may be a key metabolic pathway in the regulation of PitNETs.

KEGG enrichment analysis of the 43 core targets showed that IL-17 was a key signaling pathway in the regulation of PitNETs; the IL-17 signaling pathway also plays a key role in inflammation-related diseases (Bunte and Beikler, 2019; Chang and Dong, 2011). IL-17 signaling is known to promote gut barrier immunity via regulation of microbes as well as drive tumor growth (Chandra et al., 2024). Inflammation is closely related to the development of PitNETs (Wang et al., 2021; Chen et al., 2015). Studies have shown that the serum IL-17 levels in patients with invasive PitNETs are significantly higher than those in patients with non-invasive PitNETs (Qiu et al., 2011); furthermore, the median serum IL-17A level in patients with PitNETs is higher than that in the control group (Glebauskiene et al., 2018). CXCL8 participated in the IL-17 signaling pathway (Jain, 2022) and is known to be closely related to tryptophan metabolism. The conversion of tryptophan into kynurenine has been shown to be related to inflammation (Gustafsson et al., 2020), which indicates that CXCL8 may be a potential target for PitNET treatment. Chronic inflammation is an important factor in the occurrence and development of tumors (Coussens and Werb, 2002; Liu et al., 2016). CXCL8 is a PitNET-derived cytokine that plays an important role in the tumor microenvironment (Marques et al., 2019) to regulate tumor proliferation, invasion, and migration in an autocrine or a paracrine manner. CXCL8 can be integrated with multiple intracellular signaling pathways to produce synergistic effects (Liu et al., 2016); it is important for the activation and transport of inflammatory mediators as well as progression and metastasis of tumors (Ha et al., 2017).

*B. adolescentis* may be an important microbe related to PitNETs. A recent study showed that *B. adolescentis* produces the microbial metabolite hypaphorine, which inhibits inflammatory responses and hepatic oxidative stress (Qin et al., 2024). Furthermore, dietary supplementation with *B. adolescentis* has been shown to augment tightening of the intestinal barrier, dampened inflammatory (Roberts et al., 2020) and *B.adolescentis* can suppress tumorigenesis (Lin et al., 2023; Chen et al., 2023). Preincubation of HT-29 cells with *B. adolescentis* FRP 61 was shown to significantly inhibit CXCL8 secretion (Carey and Kostrzynska, 2013).

Herein, we found that baicalin, baicalein, and compound K have good binding activities with CXCL8. Baicalin is an important flavonoid isolated from *Scutellaria baicalensis* Georgi and exhibits important anti-inflammatory, anti-infection, and antitumor functions (Zhao et al., 2020). Baicalein is an important medicinal flavonoid derivative of *S. baicalensis* Georgi that has anti-inflammatory and anticancer properties (Gupta et al., 2022), in addition to being able to regulate the tumor microenvironment (Wang et al., 2024). Compound K is a rare protopanaxadiol type of ginsenoside that inhibits tumor growth by inducing tumor apoptosis and tumor cell differentiation; it also regulates the tumor microenvironment by inhibiting tumor-angiogenesis-related proteins (Liu et al., 2022). And compound K can be used to treat chronic inflammatory diseases (Tian et al., 2023). These findings indicate that baicalin, baicalein, and compound K could be promising effectors in the treatment of PitNETs.

Thus, this study shows that *B. adolescentis* may be an important microbe related to PitNETs and that tryptophan metabolism may be a key metabolic pathway in PitNETs treatment and it is closely related to CXCL8. In addition, the results suggest that baicalin, baicalein, and compound K may affect the IL-17 signaling pathway in PitNETs through CXCL8. These findings provide important viewpoints for further research.

As a new interdisciplinary research method, network pharmacology shows great potential in revealing the actions mechanisms, targets, and biological effects of metabolites; therefore, it has significance for comprehensively and deeply understanding the interactions between metabolites and organisms. However, there are some limitations and challenges to using this approach, including imperfect information in the databases used, incomplete data, and the need to verify the accuracies of the target predictions. Thus, in the future, we intend to further verify our current findings through experiments.

## Data Availability

The original contributions presented in the study are included in the article/Supplementary Material; further inquiries can be directed to the corresponding authors.
